# Evaluation of COVID-19 pandemic on components of social and mental health using machine learning, analysing United States data in 2020

**DOI:** 10.3389/fpsyt.2022.933439

**Published:** 2022-08-08

**Authors:** Seyed-Ali Sadegh-Zadeh, Mahboobe Bahrami, Amirreza Najafi, Meisam Asgari-Ahi, Russell Campion, Amir M. Hajiyavand

**Affiliations:** ^1^Department of Computing, Staffordshire University, Stoke-on-Trent, United Kingdom; ^2^Behavioral Sciences Research Center, School of Medicine, Isfahan University of Medical Sciences, Isfahan, Iran; ^3^Department of Information Technology Engineering, Tarbiat Modares University, Tehran, Iran; ^4^Department of Information Technology Management, University of Tehran, Tehran, Iran; ^5^Department of Mechanical Engineering, School of Engineering, University of Birmingham, Birmingham, United Kingdom

**Keywords:** mental health, COVID-19 pandemic, social behaviours, psychiatry issues, machine learning, statistic analysis, prediction model

## Abstract

**Background:**

COVID-19 was named a global pandemic by the World Health Organization in March 2020. Governments across the world issued various restrictions such as staying at home. These restrictions significantly influenced mental health worldwide. This study aims to document the prevalence of mental health problems and their relationship with the quality and quantity of social relationships affected by the pandemic during the United States national lockdown.

**Methods:**

Sample data was employed from the COVID-19 Impact Survey on April 20–26, 2020, May 4–10, 2020, and May 30–June 8, 2020 from United States Dataset. A total number of 8790, 8975, and 7506 adults participated in this study for April, May and June, respectively. Participants’ mental health evaluations were compared clinically by looking at the quantity and quality of their social ties before and during the pandemic using machine learning techniques. To predict relationships between COVID-19 mental health and demographic and social factors, we employed random forest, support vector machine, Naive Bayes, and logistic regression.

**Results:**

The result for each contributing feature has been analyzed separately in detail. On the other hand, the influence of each feature was studied to evaluate the impact of COVID-19 on mental health. The overall result of our research indicates that people who had previously been diagnosed with any type of mental illness were most affected by the new constraints during the pandemic. These people were among the most vulnerable due to the imposed changes in lifestyle.

**Conclusion:**

This study estimates the occurrence of mental illness among adults with and without a history of mental disease during the COVID-19 preventative limitations. With the persistence of quarantine limitations, the prevalence of psychiatric issues grew. In the third survey, which was done under quarantine or house restrictions, mental health problems and acute stress reactions were substantially greater than in the prior two surveys. The findings of the study reveal that more focused messaging and support are needed for those with a history of mental illness throughout the implementation of restrictions.

## Introduction

SARS-CoV-2 (SARS) originated from Wuhan, China, (1) in December 2019, breaching international borders, slowing economies around the world. COVID-19 is a contagious disease that manifests itself in a variety of ways having common symptoms such as fever, cough, exhaustion, shortness of breath, and headaches (2, 3). During the initial months of the COVID-19 pandemic, countries all over the world took extraordinary steps to stop the SARS CoV-2 virus from spreading (4). California experienced the first state-wide shutdown on March 19, 2020. Within months, the country had passed some type of limitation, with the majority of the inhabitants of the United States being asked to stay at home and restrict their physical proximity to people (5). Mapping, where United States has been shut down, “The Washington Post, March 18, 2020.” As a result, the majority of “non-essential” job operations in municipal governments terminated or migrated to remote videoconferencing or work from home possibilities. This resulted in widespread furloughs and mass unemployment for a significant portion of the population (6): United States now has 22 million unemployed, wiping out a decade of job gains “The Washington Post, April 17, 2020),” (7). There is no doubt that such efforts fundamentally altered the social and psychological aspects of a large part of the population. As a result, the pandemic’s long-term impacts and accompanying limits on cultural, social, and mental health will almost certainly be a subject of research for years ahead.

The economic repercussions of the shelter-in-place countermeasures were quickly felt across the country, followed by social and psychological consequences (8). As an example, over 33 million Americans filed new jobless claims within the first 6 weeks of state-wide stay-at-home directives, a level of job loss not seen since the Great Depression (6): United States now has 22 million unemployed, wiping out a decade of job gains (The Washington Post, April 17, 2020). In terms of mental and social health, the unprecedented increase in unemployment was concerning, given the well-documented findings that high unemployment, financial worries, and feelings of loneliness are major causes of suicide, substance misuse, domestic violence, and other mental health and social health concerns (9). The broad uncertainty about the pandemic’s probable course, as well as broad concerns about health and economic instability as a result of the lockdown restrictions, has raised fears that a boom in mental health issues is on the future (10, 11). The concern about the disease, its transmissibility, as well as its mortality led to panic and generalized anxiety, raising concerns that, as in other nations, post-traumatic psychosocial stressors could last long after the pandemic ended (12, 13).

Prior disease outbreak quarantines have been demonstrated to greatly increase manifestations of post-traumatic stress disorder and depression in the general population (14–16). Furthermore, extended stay-at-home mandates and social distancing measures might have unanticipated mental health repercussions, as they limit many of the aspects of everyday living that contribute to emotional strength, socialization, and life pleasure (17, 18). Because of the scale of the pandemic and its impact on daily life, many experts are concerned that mental health issues will continue to be a problem for years to come, with a negative impact on society and culture (19, 20). As a result, it appears that successful recovery from the outbreak will necessitate a thorough knowledge of the mental health repercussions that developed during the crisis’ severe stages.

Several research studies show that the pandemic could have a negative impact on people’s mental health (20–28). The COVID-19 pandemic’s stressors and motives to practice social distancing appear to be difficult for people to understand, resulting in poor mental health outcomes (20). Negative coping abilities, which are risk factors for depression, stress, and trauma among people of all ages, contribute to the inefficient ability to process stressful conditions like the pandemic (29). Another important component in determining people’s mental resilience during times of crisis, such as the pandemic, was social support. During the pandemic, people felt high levels of low to moderate social support, which contributed to rises in anxiety and despair (30). Another risk for people’s mental health during the pandemic was addiction (31). Despite being obliged to stay at home throughout the pandemic, some were found to be still using drugs. During the pandemic, people increased their use of alcohol and cannabis, with 49.3% engaged in drug use alone (29).

We looked at the prevalence of mental health disorders in a nationally representative sample of individuals in the United States, taken right at the start of the pandemic, during the first months of countrywide stay-at-home regulations. In this sample of data, we analyzed social relations and mental health concerns to identify the differences in mental health outcomes related to the lockdown. These data and analyses will be crucial in recording mental health and its impact on society during the early stages of the COVID-19 outbreak, as well as serving as a guideline for future studies on the crisis’ long-term psychiatric effects.

## Materials and methods

### Dataset description

Data Foundation’s national COVID-19 Impact Survey was used in this study (32). It provided data from the COVID-19 Impact Survey, which gives statistics on physical and mental health, economic security, and social dynamics in the United States as a result of the coronavirus pandemic. The probability-based survey, performed by National Opinion Research Center (NORC) at the University of Chicago for the Data Foundation, gives estimates for the entire United States, along with 10 states and eight urban regions. California, Colorado, Florida, Louisiana, Minnesota, Missouri, Montana, New York, Oregon, and Texas are among the states, while Atlanta, Baltimore, Birmingham, Chicago, Cleveland, Columbus, Phoenix, and Pittsburgh are among the urban regions. Data was collected in 3 phases (April, May, and June, 2020) to provide a picture of the global pandemic’s impact on physical and mental health, economy, and employment in the United States during each phase. The three phases are a series of distinct cross-sectional investigations. Data was collected over the course of a week for each wave, with interviews done in both English and Spanish. Firstly, a random sample of United States homes was drawn from the non-partisan and impartial research organization NORC at the University of Chicago National Sample Frame and then contacted via mail, email, telephone, and field interviewers in the United States. People having merely a P.O. Box addresses were not included in the USPS Delivery Sequence File, and certain recently built homes were excluded from the study. A participant was chosen at random from each household who lived with one or more than one adult housemate (family, friend, partner). The survey was available to all requested members online or by telephone with a NORC telephone researcher. The dataset is intended to provide a continuous assessment of the public’s view, health, and economic situation during the outbreak to see how things are changing. When many pieces of information are available, it will be possible to track how concerns such as COVID-19 signs and financial status evolve over time. Physical health, economic and financial health, and social and mental health are the three main research areas covered by the survey. Mental health, work from home, communication, COVID-19 symptoms, chronic medical issues, behavioral components, and many more indicators were included in the survey questions. For this study, data from weeks 1 (April 20–26, 2020), 2 (May 4–10, 2020), and 3 (May 30–June 8, 2020) were available and integrated. The following are the five psychosocial questions we look at:

1. Felt nervous, anxious, or on edge?
2. Felt depressed?
3. Felt lonely?
4. Felt hopeless about the future?
5. Had physical reactions such as sweating, trouble breathing, nausea or a pounding heart when thinking about your experience with the coronavirus pandemic?

The following were the response options:

1. Not at all or less than 1 day
2. 1–2 days
3. 3–4 days
4. 5–7 days

Each answer was given a value of zero, one, two, or three, depending on the question. The mean score for questions 1, 2, 3, and 4 is 0.64 (SE = 0.01), whereas the mean score for question 5 is 0.15 (SE = 0.01).

### Reliability and independency

In this study, a model was built for mental health markers to demonstrate the links between the attributes and the symptoms. The data were collected over the course of 7 days. A total of five attributes were considered for this which were nervousness, anxiety, depression, feeling lonely and feeling hopeless while four symptoms have been linked as the outcome of these attributes which were sweating, trouble breathing, pounding heart, and other symptoms. For this, an item reliability analysis was conducted to assess the consistency of responses to the mental health questions. This study constructed Cronbach alpha, a scale for quantifying the reliability of internal consistency. Following that, a pairwise chi-square test of independence was added to look for correlations between mental health markers and other variables, with a *P*-value of 0.05 as the significance limit.

### Machine learning techniques

Many media outlets today use the phrase “we live in the information age,” but many scholars feel we are living in the data age. We live in a world where a massive amount of data is generated every day in a variety of disciplines. As a result, these data can be applied to a variety of sectors, including mental health. Because of the vast volume of this data, typical data analysis methodologies and tools are frequently unavailable, making extracting information from it difficult. We are often unable to apply these approaches due to the differences between new data and old data, as well as the lack of responses to traditional queries; as a result, new ways, including the concept of machine learning, are required. Machine learning is a branch of research that enables machines to learn without having to plan ahead of time. Machine learning is the study of how computers learn from data and improve their performance. The main focus of the study is on automated learning and the detection of complicated patterns in order to create a system that can make intelligent data-driven decisions. The sorts of machine learning systems will be discussed in the following sections. Based on the amount and type of supervision they receive during training, machine learning systems can be divided into four categories, i.e., supervised learning, unsupervised learning, semi-supervised learning, and reinforcement learning.

Supervised learning was employed in this study. Each pattern in the set intended for teaching the algorithm has a label that represents the expected output in supervised learning. In supervised learning, there is a range of algorithms from which to choose, and four of them were utilized for the purpose of comparison in this study. The random forest algorithm is one of the ensemble methods that use a set of tree algorithms to conduct classification operations. To train each decision tree and, eventually, make decisions, this system employs data sets. The SVM algorithm produces decisions by determining the optimum data border that is the furthest away from all other categories (their supporting vectors). The logistic regression algorithm is determined using a class estimate function of each instance in the Naive Bayes algorithm, which calculates the likelihood of membership in each class using Bayesian theory. Hyperparameters in each of these models must be fine-tuned. To fine-tune these models, we used the grid search method. By experimenting with different parameters, this strategy aids in improving the model’s accuracy. Two sets of training and test data are needed to evaluate these strategies. This data is utilized as training data for 80% of the time and as test data for 20% of the time. It is then put to use based on a variety of parameters. The accuracy criteria look at the model’s performance across all classes. When the relevance of the classes is equal, this criterion is important. The accuracy of the model in the presence or absence of circumstances is measured by sensitivity and specificity. These criteria have been chosen due to the relevance of decision making, assuming equal value of the classes and a greater importance of one of the classes.

### Background on graph and network

Graph and network science are one of the fields that plays a major role in data science. Graphs were used to assess the reaction of several features in this study. Each feature’s answers were taken to be 90 and the graph’s edges to be pairs of responses that occur together. Because of the greater illustration and understanding of the relationships between the offered responses, this method was chosen. Some graph-related metrics, such as centrality, were calculated to determine the effect of each of the solutions. The centrality betweenness and closeness indices will be used to divide this calculation. The degree to which a response is in the shortest path between responses is measured in betweenness, while the proximity of each response to the other responses is measured in closeness. In general, each answer with a higher centrality index value is more essential. To create graphs and calculate various metrics, the Python programming language and Gephi software were utilized. The data was then processed using Python and the relevant data science packages. A subset of characteristics due to the model’s complexity was selected and a vast amount of data features, as well as its correctness. Effective features have been exploited using Markov blanket.

## Results

The results were collected over three months and every month is demonstrated separately. The total number of 8,769, 8,952, and 7,491 participants contributed to the survey in April, May and June, 2020 respectively. In this article, the working age is considered to be between 25 to 65 years old. This contains around 60% of participants. The average annual income in the United States is c.$50K and this was the case for 60% of participants averaged over three months. 13,233 (52.5%) participants had a bachelor’s degree or more. During the course of these three months, almost 30% of the total participants were living alone, while around 33% of the total number lived at least with one adult. On the other hand, c.24% lived with at least one child. Table 1 indicates the detailed analysis of the collected data.

**TABLE 1:** The study attributes and the participation population.

Description	April 2020	May 2020	June 2020	Total
Total participant	8,769	8,952	7,491	25,212
Working age prior retirement 25 to 65 years old	5,843 *(66.6%)*	5,878 *(65.6%)*	4,919 *(65.6%)*	16,640 *(66.0%)*
Income $50k	5,274 *(60.1%)*	5,410 *(60.4%)*	4,324 *(57.7%)*	15,008 *(59.5%)*
Education (minimum bachelor’s degree)	4,626 *(52.7%)*	4,722 *(52.7%)*	3,885 *(51.8%)*	13,233 *(52.5%)*
Living alone	2,626 *(29.9%)*	2,761 *(30.8%)*	2,324 *(31%)*	7,711 *(30.6%)*
Living with kids	2096 *(23.9%)*	2093 *(23.4%)*	1807 *(24.1%)*	5996 *(23.8%)*
Adults only – no kid	5645 *(64.3%)*	5830 *(65.1%)*	4745 *(63.3%)*	16220 *(64.3%)*

**FIGURE 1:**
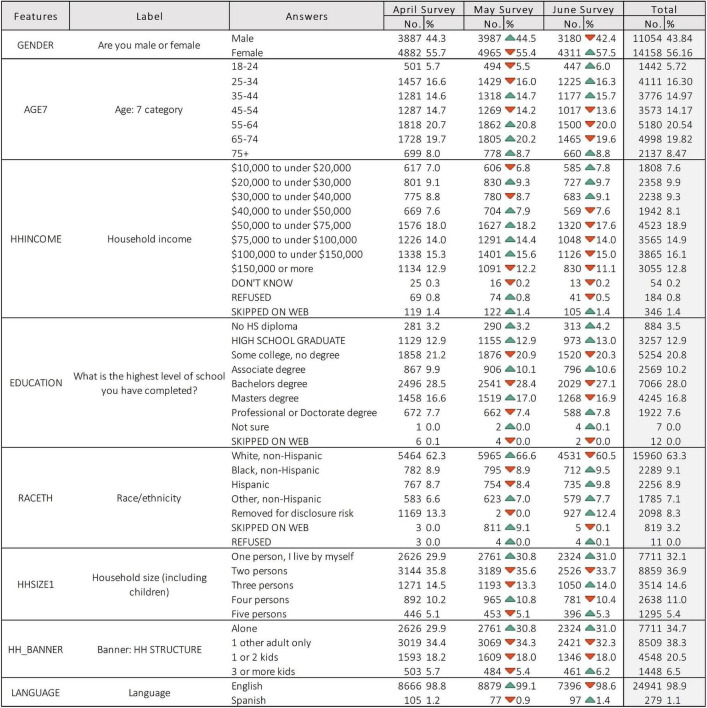
Demography of COVID-19 impact survey participants.

In this dataset, the respondents raised a concern regarding one or more of the mental health markers. In terms of mental health markers, 37.60% (9,481) of respondents reported nervousness, anxiety, or being on edge. 38.22% (9,639) reported depression, 37.95% (9,571) reported loneliness, and 38.02% (9,588) felt hopeless about the future. Out of this report, only 9.62% (2,425) declared at least one physical reaction such as sweating, trouble breathing, nausea or a pounding heart during the coronavirus pandemic. The social demographic features of dataset participants are depicted in Figure 1.

### Statistical results

This section reports the statistics regarding the impact of the COVID 19 pandemic on mental health of members of society by incorporating experiments and factors linked to the impact of COVID 19, such as the influence on social and cyberspace interactions and volunteer activities. In this study, the links between the various factors such as gender, age, income, education, race, and the level of trust in people such friends, families, neighbors, etc. were analyzed.

For this study, eight features were extracted from the dataset to conduct the Statistical Experiments which show the differences in behavior before and after the COVID-19 pandemic. The following list indicates the questions which will then be used for correlation to the comparisons shown in Table 2:

**TABLE 2:** Statistical experiments.

Number	Description	Associated questions
SE01	Changes in social relationships before and after the pandemic	SOC2A and SOC2B
SE02 SE04	Changes in age, income, education	SOC2A and SOC2B SOC3A and SOC3B
SE03	Changes in the use of virtual relationships before and after the pandemic	SOC3A and SOC3B
SE05	Change in participation in voluntary activities before and after COVID 19	SOC4A and SOC4B
SE06	Changes in age, income, education and Changes in the use of virtual relationships before and after the pandemic	SOC4A and SOC4B
SE07	Changes in job status of people before and after pandemic	ECON3, ECON4A, ECON4B

• SOC2A: In the past month, how often did you talk with any of your neighbors?
• SOC2B: During a typical month prior to March 1, 2020, when COVID-19 began spreading in the United States, how often did you talk with any of your neighbors?
• SOC3A: In the past month, how often did you communicate with friends and family by phone, text, email, app, or using the Internet?
• SOC3B: During a typical month prior to March 1, 2020, when COVID-19 began spreading in the United States, how often did you communicate with friends and family by phone, text, email, app, or using the Internet?
• SOC4A: In the past month, did you spend any time volunteering for any organization or association, or not?
• SOC4B: During a typical month prior to March 1, 2020, when COVID-19 began spreading in the United States, did you spend any time volunteering for any organization or association, or not?
• ECON4A: Think about 30 days from now, how likely do you think it is that you will be employed at that time?
• ECON4B: Think about 3 months from now, how likely do you think it is that you will be employed at that time?

In analyzing each experiment, a heat map diagram is demonstrated to compare the correlation between the features and a histogram of the variables for all three surveys is presented separately for each survey.

A variety of statistical tests was used to assess the correlations between variables in order to analyze the influence of the COVID-19 pandemic on participants’ socio-mental features. The H0 hypothesis was rejected in all experiments using Pearson’s chi-squared test.

**FIGURE 2:**
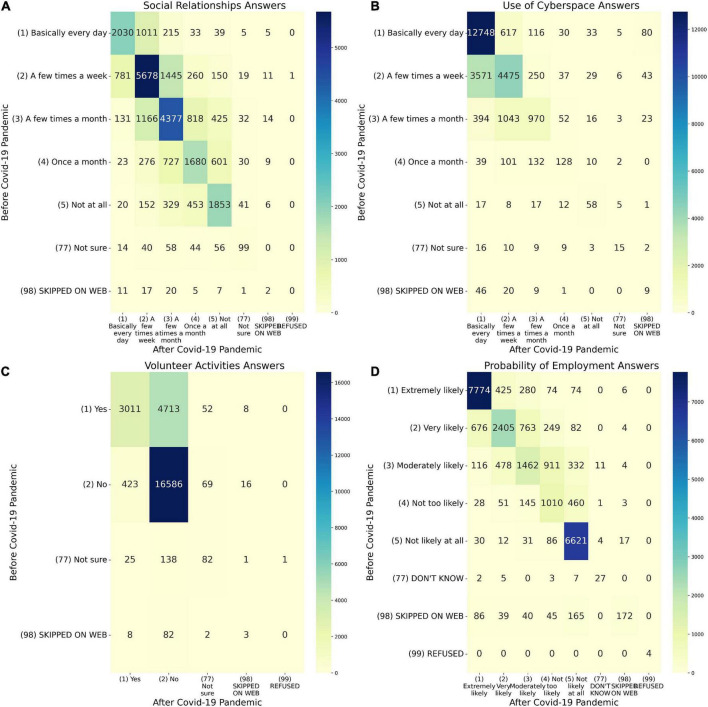
Heatmap analysis of the variables in Experiments. **(A)** Heatmap of social relationships answers between before COVID-19 pandemic (SOC2B) and during last month (SOC2A). **(B)** Heatmap of use of cyberspace answers between before COVID-19 pandemic (SOC3B) and during last month (SOC3A). **(C)** Heatmap of volunteer activities answers between before COVID-19 pandemic (SOC4B) and during last month (SOC4A). **(D)** Heatmap of probability of employment answers between before COVID-19 pandemic (ECON4B) and during last month (ECON4A).

As indicated in Figure 2A, SE01 analyses the “conversations with relatives” attribute before and during the pandemic. A total of 25,000 respondents replied to this, and around 15,400 (60%) of them did not change their conversation time before and during the Pandemic.

The sum of the numbers above the diameter heat map indicates that 5,200 people reduced their time spent talking to relatives while 4,200 increased their conversation time. The correlation between the responses is demonstrated as a normalized distribution, which is a value between zero and one. Cells with higher positive numbers imply more correlation, where lower negative numbers suggest that the response event is contradictory, and values near zero show that the response event is independent.

**FIGURE 3:**
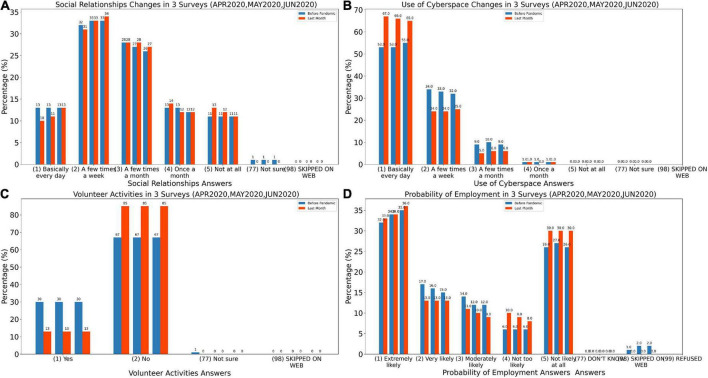
Histogram of analysis of the variables in Experiments. **(A)** Histogram of social relationships answers for before COVID-19 pandemic (SOC2B) and during last month (SOC2A). **(B)** Histogram of use of cyberspace answers for before COVID-19 pandemic (SOC3B) and during last month (SOC3A). **(C)** Histogram of volunteer activities answers for before COVID-19 pandemic (SOC4B) and during last month (SOC4A). **(D)** Histogram of probability of employment answers for before COVID-19 pandemic (ECON4B) and during last month (ECON4A).

Figure 3A demonstrates general behavior of social relationships over three months and compares the relationship between the period before the pandemic (indicated in blue) and during pandemic (indicated in red). The results show that almost 33% of the population did some networking during the week but only 28% of participants networked several times a month.

**FIGURE 4:**
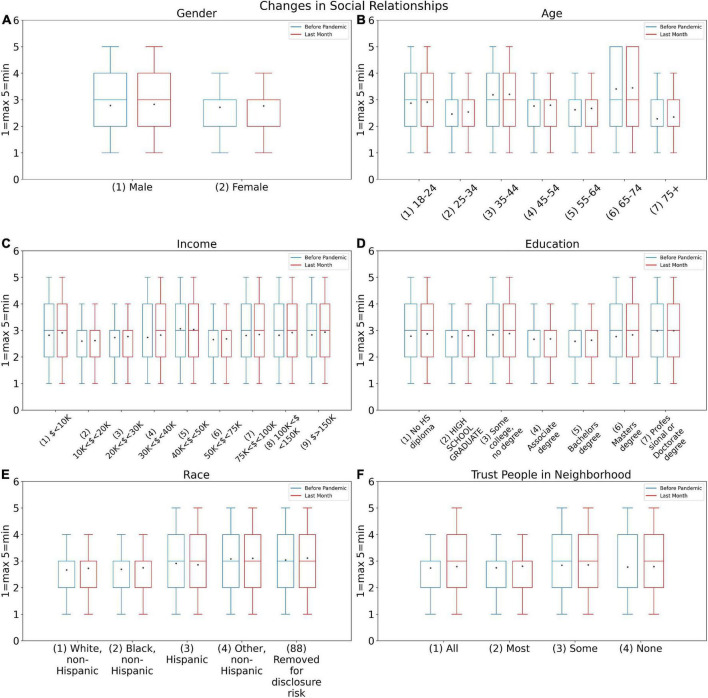
Changes in social relationships answers for before COVID-19 pandemic (SOC2B) and during last month (SOC2A) based on **(A)** Gender, **(B)** Age, **(C)** Income, **(D)** Education, **(E)** Race, and **(F)** Trust people in neighborhood.

Figure 4 demonstrates the results obtained from the SE02 test, which examines how age and gender, income, education, race, and participants’ trust affected their intention to talk with relatives. In the figure, the blue cells depict the statistical distribution of replies prior to the COVID-19 pandemic, whereas the orange cells depict the statistical distribution of responses during the COVID-19 pandemic. The numbers 1 to 5 on the vertical axis of the answers reflect the responses to the conversation question, where 1 indicates the maximum number of discussions (daily conversation) and 5 indicates the lowest number of interactions (do not make any conversation). The dot on the box also represents the average of the responses. It is predictable that no alteration would be reported for ‘age’, ‘gender’ and ‘race’ attributes. However, the results showed that the other attributes have also no changes.

The graphs indicated fewer variations to the mentioned attributes before and during the pandemic. However, they provide valuable information regarding the differences in communication behavior of different genders and also different age groups. Based on this, women have broader social relationships than men. On the other hand, people in the age group 18–24 years old, 35–44 years old, and 65–74 years old indicated more social relationships before pandemic than the other age groups. This then indicates that the pandemic has had a greater impact on these particular group and has affected their relationships.

The impact of people’s income on conversations is investigated in Figure 4C. The scale of averages for middle-income persons is the only interesting element, since it reveals that their talks have stayed consistent since the outbreak. However, the pandemic has reduced the number of conversations in the other categories, and the average after the outbreak is greater than before the pandemic. Figure 4D depicts the level of education and the amount of conversations. As is well known, those with higher education (bachelor’s, master’s, and Ph.D.) had more talks both before and after the pandemic than others, and they were unable to entirely restrict their interactions. Furthermore, the average remains consistent for individuals without a diploma or schooling, demonstrating that those with a very low level of education have not limited their talks following the pandemic. Other groups, on the other hand, have more limited conversations. Figure 4E demonstrates that whites were less constrained in their interactions after the pandemic, while blacks had a lower mean, indicating an increase in their conversations after the pandemic began. Figure 4F depicts the effect of individual trust, demonstrating that people who trusted others had fewer ties, whilst persons who had no or little trust in others did not have much of a pandemic effect.

The link between the two features SOC2B and SOC2A is studied in the SE03 experiment which is demonstrated in Figures 2 and 3. Figures 2B is a heat map which reveals the majority of participants are using cyberspace. Unlike the previous heat map, the total of the numbers below the chart’s diameter is more than the top diameter, indicating that people are increasingly using cyberspace and that the number of people who have increased their usage of cyberspace has been raised.

The histogram in Figure 3B demonstrates that the number of people who use cyberspace on a daily basis has significantly increased in all three surveys since the beginning of the pandemic. This is even more significant where the use cyberspace happens on a daily basis since the beginning of the pandemic. As it is shown, the growth rate was higher in the first survey and decreased in the second and third polls. For this particular attribute, it is essential to consider the level of knowledge in using cyberspace and additionally the link between the age- and job-related factors.

**FIGURE 5:**
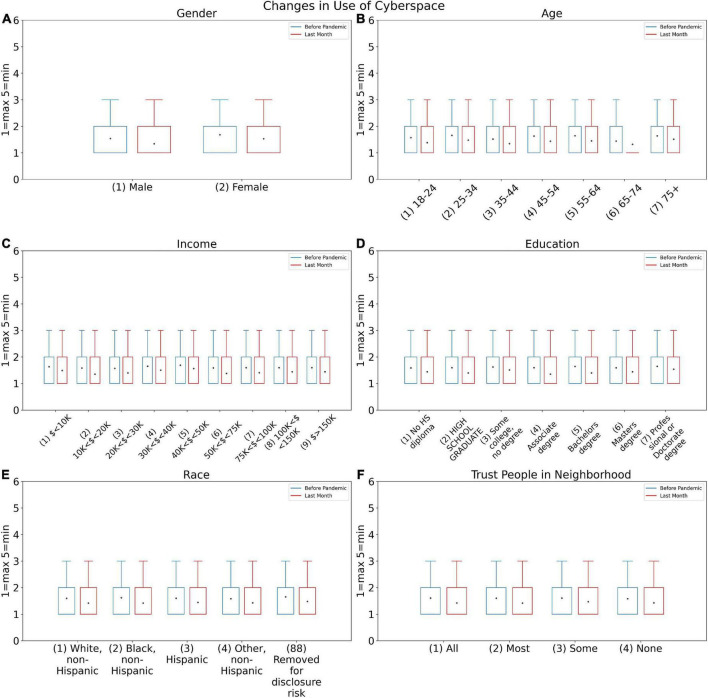
Changes in use of cyberspace answers for before COVID-19 pandemic (SOC3B) and during last month (SOC3A) based on **(A)** Gender, **(B)** Age, **(C)** Income, **(D)** Education, **(E)** Race, and **(F)** Trust people in neighborhood.

The histogram in Figure 3B demonstrates that the number of persons who use cyberspace on a daily basis has increased in all three surveys since the beginning of the pandemic. As illustrated in Figure 5, in general, the use of Cyberspace increased among all attributes during the pandemic. Figure 5A shows that women had generally a lower average of cyberspace users than men. However, both women and men are using cyberspace more than they were using it before the pandemic. Figure 5B shows that the age group between 15 and 24 years was the most engaged in cyberspace during the pandemic, however, as age increased, the use of the cyberspace decreases. Similar to before, this usage also increased during the pandemic which was because of being in lockdown. Figure 5C shows that there is no link between income and cyberspace usage.

In the SE05 Experiment, it is evident that the number of people who have quit volunteering since the pandemic has dramatically increased, according to the heat map in Figure 2C. On the other hand, as shown in Figure 3C, the intention of having volunteer activities significantly decreased during the pandemic. This may be due to the fear of COVID-19 or because of being locked down.

**FIGURE 6:**
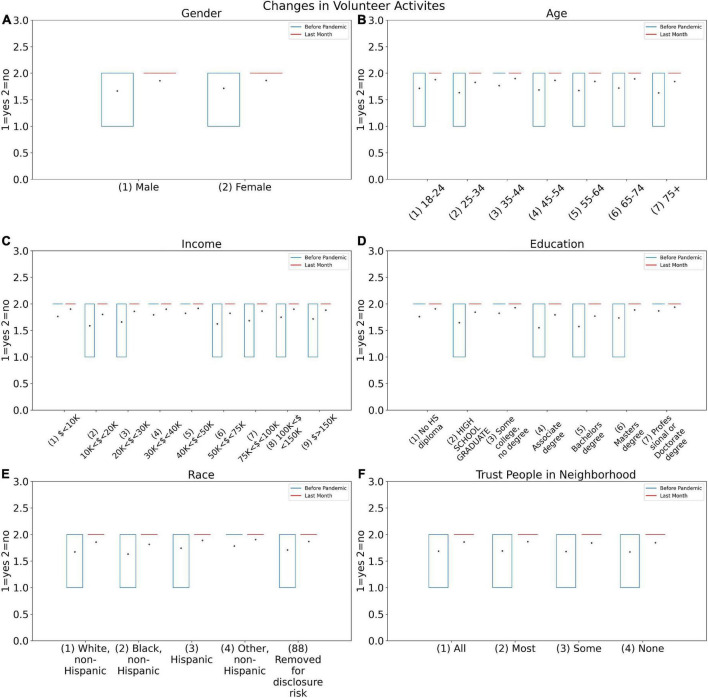
Changes in volunteer activities answers for before COVID-19 pandemic (SOC4B) and during last month (SOC4A) based on **(A)** Gender, **(B)** Age, **(C)** Income, **(D)** Education, **(E)** Race, and **(F)** Trust people in neighborhood.

It was a general habit for the majority of the age groups to volunteer for certain jobs (this was less for the age group 25–34 years old). However, during the pandemic, both men and women declined to be volunteers, although women declined more (Figure 6). The volunteering of higher educated people has also decreased during the pandemic. The reason would be either a drop in the supply of volunteering work due to lockdown or it may again be fear of catching the virus. Regardless of the reason, this caused people to stay more indoors and socio-communication was decreased.

Figure 2D demonstrates the results from the SE07 Experiment which shows that most people did not notice a change in their employment prospects. Furthermore, the numbers above the diameter are higher than the numbers below the diameter, implying that people believe their chances of finding work during the pandemic have reduced. Figure 3D demonstrates the histogram of the probability of employment during the three months. The motivation of applying for jobs remained similar before and during the pandemic. However, it is shown that people’s hope regarding their chances of finding work had declined during the pandemic.

For data consistency evaluation, the Cronbach’s Alpha measure was calculated for each experiment. The results in Table 3 illustrate the reliability of over 0.90 which demonstrates the highly reliable data as it was over 0.7 threshold. These experiments support the changes in social activities, use of cyberspace, volunteer activities and employment probability in relation to gender, age, income, education, race and trust on people, all before and during pandemic.

**TABLE 3:** Reliability analysis results (Cronbach’s Alpha measure).

Reliability test	Gender	Age7	Hhincome	Education	Raceth	SOC1
SOC2B, SOC2A	1	0.994	0.993	0.994	0.999	0.999
SOC3B, SOC3A	1	0.992	0.973	0.955	0.999	0.999
SOC4B, SOC4A	1	0.965	0.988	0.978	0.999	0.999
ECON4B, ECON4A	1	0.909	0.955	0.954	0.999	0.999

In this study, a pairwise Pearson chi-square examination was conducted to evaluate the association between various variables, where P values were assigned to be less than 0.05 as shown in Table 4. Based on the independency examination of the data, it was found that there is no relation between the trust of the participants (SOC1) and the level of the volunteer activities (SOC4A and SOC4B) as it was expected.

**TABLE 4:** Independency analysis – Pearson Chi-Square test.

Independency Test	*P*-value			
	SOC2B, SOC2A	SOC3B, SOC3A	SOC4B, SOC4A	ECON4B, ECON4A
Gender	5.62 × 10^–28^ Reject H0	1.17 × 10^–10^ Reject H0	6.66 × 10^–21^ Reject H0	3.13 × 10^–9^ Reject H0
Age7	7.10 × 10^–5^ Reject H0	2.92 × 10^–7^ Reject H0	1.14 × 10^–11^ Reject H0	2.19 × 10^–203^ Reject H0
Hhincome	4.48 × 10^–12^ Reject H0	1.58 × 10^–18^ Reject H0	2.10 × 10^–34^ Reject H0	1.52 × 10^–68^ Reject H0
Education	3.52 × 10^–7^ Reject H0	3.52 × 10^–39^ Reject H0	1.09 × 10^–71^ Reject H0	1.08 × 10^–54^ Reject H0
Raceth	4.85 × 10^–19^ Reject H0	9.18 × 10^–10^ Reject H0	1.38 × 10^–^12 Reject H0	9.96 × 10^–34^ Reject H0
SOC1	3.26 × 10^–4^ Reject H0	9.03 × 10^–3^ Reject H0	0.80 (H0 holds true)	2.83 × 10^–3^ Reject H0

### Machine learning results

Graph and network science is one of the fields that has recently played a major part in data science. Graphs were used to assess the reaction of several features in this study. The responses to each of the features are treated as nodes in this section, and the edges of the graph are treated as pairs of replies that occur together. Some graph-related metrics, such as centrality, were calculated to determine the effect of each of the responses. The betweenness and closeness centrality metrics will be used to divide this calculation. Betweenness represents how short the path is from one response to all other responses, and closeness represents how closely responses to each other on average. In general, each answer with a higher centrality index value is more essential. To construct graphs and calculate various criteria, Python and Gephi were utilized.

Each answer with a higher centrality index score is, in general, more essential. We need to pick a subset of features due to the model’s complexity and a large number of features, as well as its accuracy. Effective features in psychological conditions have been picked from this dataset based on the research [14] utilizing the Markov blanket. 80% of this data is used as training data and 20% as test data. The Synthetic Minority Oversampling Technique (SMOTE technique) was used to address the class imbalance. Some data were recognized as outliers and eliminated from the dataset under the supervision of a fellow psychiatrist. Finally, several supervised learning approaches such as random forest, support vector machine, Naive Bayes, and logistic regression have been applied to predict mental health using the Python and the Scikit-learn package.

**FIGURE 7:**
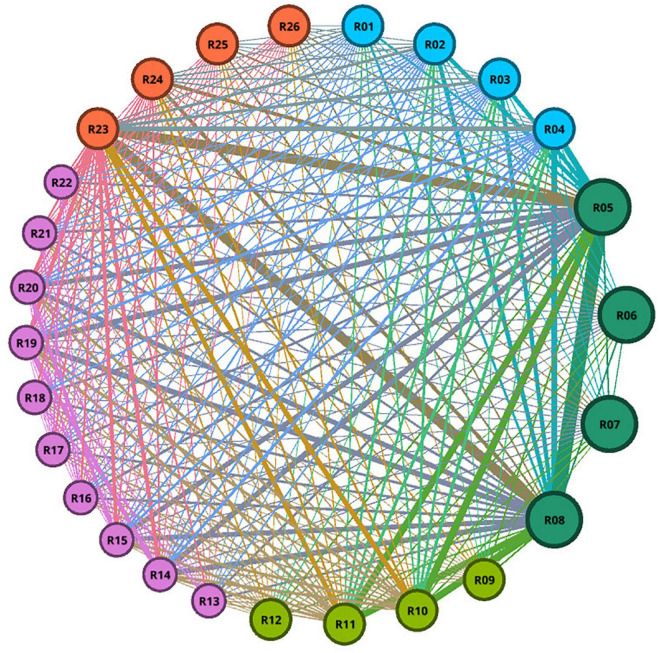
For all responders, a graph of selected feature responses is shown. The thickness of the lines indicates the strength of pairwise responses relations; a thicker association between two nodes indicates a stronger relationship. The size of graph nodes is determined by how connected they are to other answers. The same color was used to group nodes based on their closeness and betweenness centrality.

**FIGURE 8:**
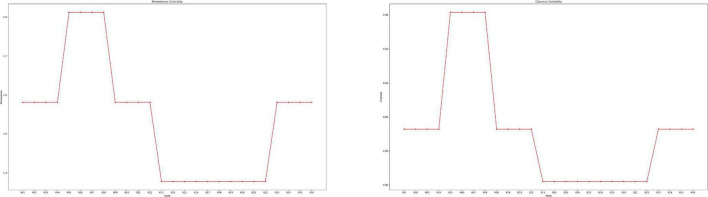
The importance of responses will be determined using the betweenness centrality metrics. Betweenness is a measure of how short the road between one reaction and all others is. Each answer with a higher centrality index score is, on average, more important. The importance of responses will be determined using the closeness centrality measures. Closeness refers to how similar responses are on average. Each answer with a higher centrality index score is, on average, more important.

The graph in Figure 7 is made up of nodes representing the selected attributes from Table 1 and edges representing the association between pairs of responses that happened together. The relationship between the provided responses is well illustrated on the graph. Ninety edges are the names for the two basic components that make up each graph. A problem’s actors are introduced as nodes, and the relationships between those actors are introduced as edges. In order to create the graph for this study, all of the data related to the features that were chosen as nodes was first retrieved. After that, focus has been placed on the responses that occurred simultaneously in order to extract the edges. In a graph, the strength of the link can be determined by the thickness of the edges; hence, in this graph, the thickness of the edges reflects the frequency of a pair of replies. How related they are to other replies affects the size of graph nodes. Nodes were grouped according to their closeness and betweenness centrality using the same color. The color of each node based on the betweenness, and closeness is shown in Figures 8A,B, respectively. In these diagrams, the centrality of each node is calculated to determine its relevance. According to the graphs R05, R06, R07, and R08 had the highest scores among all responses. All node scores are evident, and the nodes were partitioned into groups of the same hue based on these ratings. The number of repetitions of each feature determines the thickness of each edge. The strongest feasible connection is formed between nodes R05 and R08, as indicated in the network. People in this connection have not had a fever, chills, or excessive sweating when staying at home. In the next category of strong connections, the connection between R05 and R23 as well as the connection between R08 and R23 is clear. Graph nodes are sorted by size based on how connected they are to other responses. The same color has been used to organize nodes based on betweenness and closeness centrality. As a result, nodes R05, R06, R07, and R08, which indicate whether or not to stay at home and whether or not a fever, chills, or heavy sweating, are significant.

#### Mental health prediction model experiment

To predict mental health, we employed the soc5 feature as the label of the dataset. Age (age4), physical symptoms in the preceding 7 days (phys7_4), remaining at home (phys2_18), previous clinical diagnosis of mental health status (phys3h), level of neighborhood trust (soc1), and level of a verbal conversation with neighbors (soc2a and soc2b) were also used.

Three possibilities are considered for modeling based on the soc5 feature:

1. Psychological problems less than one day a week (zero class) and psychological problems more than 5 days in a week (class one).
2. Psychological problems less than one day a week (zero class) and psychological problems more than 3 days in a week (class one).

Psychological problems less than one day a week (zero class) and psychological problems more than 1 day in a week (class one).

Random Forest, SVM, Naive Bayes, and Logistic regression are the four different Machine Learning models which were utilized to evaluate the accuracy, Sensitivity, Specificity, and Receiver operating characteristic (ROC). These ML models were employed to compare the results for each scenario. Table 5 illustrated the results of these evaluations in April, May and June, 2020, respectively. The data split 80% of the data randomly for training, and 20% of the data were randomly assigned for Testing separately for each month. Each table is divided into three main sections based on the number of occasions that psychological problems occurred during the week. In Table 5, the maximums are indicated in green and the minimums are indicated in red. As it is illustrated in Table 5, the Random forest model contributes toward the highest obtained results for the majority of the accuracy, Sensitivity, Specificity and ROC. On the other hand, Logic Regression contributes the lowest number in achieving the maximums in the evaluating factors.

**TABLE 5:** Model evaluation for all dataset.

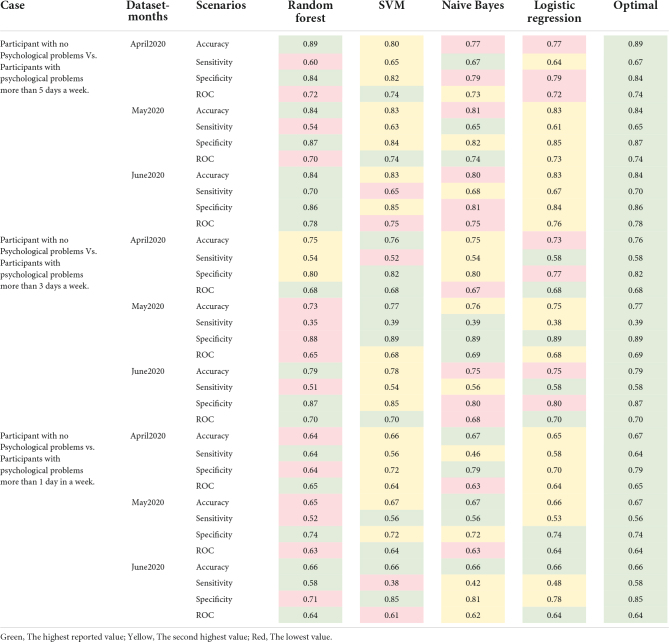

As can be seen in Tables 2 and 6, in the first possibility, i.e., the riskiest cases of mental problems, the random forest model performed better in terms of accuracy and specificity than other methods. The SVM model, on the other hand, has performed better in the Sensitivity and ROC criteria. The Logistic Regression and Naïve Bayes models were also accurately performed. On the other hand, it can be seen that the performance of the models based on accuracy has significantly decreased from the first possibility, i.e., the riskiest cases, to the last possibility, i.e., the least risky cases.

**TABLE 6:** Nodes label for responses.

Response	Label
R01	Age 18–19
R02	Age 30–44
R03	Age 45–59
R04	Age 60+
R05	Stayed home
R06	Didn’t Stayed home
R07	Felt any of (hot or feverish, chilly or cold or had chills, Been sweating more than usual) in the past 7 days
R08	Didn’t felt any of (hot or feverish, chilly or cold or had chills, Been sweating more than usual) in the past 7 days
R09	Trust All people in your neighborhood
R10	Trust most people in your neighborhood
R11	Trust some people in your neighborhood
R12	Didn’t trust people in your neighborhood
R13	Basically every day talk with your neighbors
R14	A few times a week talk with your neighbors
R15	A few times a month talk with your neighbors
R16	Once a month talk with your neighbors
R17	Not at all talk with your neighbors
R18	Typical month prior to March 1, 2020, when COVID-19 began spreading in the United States, Basically every day talk with your neighbors
R19	Typical month prior to March 1, 2020, when COVID-19 began spreading in the United States, A few times a week talk with your neighbors
R20	Typical month prior to March 1, 2020, when COVID-19 began spreading in the United States, A few times a month talk with your neighbors
R21	Typical month prior to March 1, 2020, when COVID-19 began spreading in the United States, Once a month talk with your neighbors
R22	Typical month prior to March 1, 2020, when COVID-19 began spreading in the United States, Not at all talk with your neighbors
R23	In the past 7 days, Not at all or less than 1 day Felt nervous, anxious, or on edge
R24	In the past 7 days, 1–2 days Felt nervous, anxious, or on edge
R25	In the past 7 days, 3–4 days Felt nervous, anxious, or on edge
R26	In the past 7 days, 5–7 days Felt nervous, anxious, or on edge

## Discussion

Mental health has significant social and cultural impacts. Mental disorders and suicide-related consequences have increased dramatically in all age groups and genders during the previous decade (33, 34). COVID-19’s rapid growth caused governments around the world to close public meeting places, restaurants, universities, schools, and businesses. Social isolation, digital communication, and working and educating from home have all become the new normal, and many jobs have been lost as a result. This has resulted in a high degree of anxiety, tension, and depression over the world. No studies that used modeling to not only estimate but also to describe the nuanced impacts of life events on mental health and followed by society and culture were found. The most clear finding of the research was that people who had previously been diagnosed with any type of mental disease were the most vulnerable to mental illness during the COVID-19 pandemic. As a result, governments should develop national-level programs to track the mental health of this target demographic on a regular basis and treat them appropriately.

As previously stated, the purpose of this study is to look into the impact of the COVID-19 pandemic on many aspects of people’s social conditions and mental health status. These factors include the number of genuine social contacts, the extent to which people utilize social-media, and the extent to which people participate in volunteer activities and feeling of job insecurity. The goal of examining these criteria is to determine the pandemic’s relative influence on people’s mental health. Each of these factors on its own can indicate a trend of lifestyle changes and possibly a sign of changes in a person’s mental health status during the COVID-19 pandemic. Measurement of the pattern that analyses these changes in various groups needs to be divided by gender, education level, income, age group, and race, to identify sectors of society that are more vulnerable in the pandemic. This issue aids in the development of tailored prevention and treatment protocols for certain groups. For example, a study that looked at the effect of social distance on anxiety, depression, and stress in Brazilian students found that disturbed sleep was a risk factor for mental health problems and that physical activity during quarantine was a protective factor to prevent mental health problems (35). Another study examined suicidal possibility in university students during a pandemic and showed that the student population is a vulnerable group in this regard (36).

Anxiety symptoms were reported by 37.60 percent of respondents when it came to mental health markers. When asked about their experiences with the coronavirus pandemic, 38.22 percent had depression symptoms, 37.95 percent felt lonely, 38.02 percent felt gloomy about the future, and 9.62 percent had bodily reactions such as sweating, difficulty breathing, nausea, or a racing heart. Anxiety and depression symptoms were the most common mental health disorders during this period, according to numerous recent researchers in the field of the COVID-19 pandemic, which was also the case in this study. However, when the percentage of people with physical symptoms is compared to the percentage of people with mental health problems, it becomes clear that not all of these psychiatric issues are related to physical symptoms. As a result, the involvement of social pressures and changes in people’s lifestyles in the development of mental health problems becomes clearer.

As illustrated in Figure 2, most people’s actual social interaction patterns and daily talks (62.6%) were unaffected by the pandemic and showed no change. People who altered their social communication patterns throughout the pandemic, on the other hand, gradually reduced their daily discussions with others, implying that overall social engagement declined during the pandemic. The findings suggest that roughly 4,058 persons (16.3% of the population) who are affected by the pandemic have fewer daily social contacts. This decrease could be the consequence of people avoiding contact owing to severe fear, or it could be the effect of over-adherence to protocols. Under the impact of the COVID-19 pandemic, they may also experience generalized anxiety, which has resulted in social isolation. During the COVID-19 pandemic, almost 4,997 people (20% of participants) demonstrated an increase in interpersonal social activity. This increase in social relationships through communication with others may provide an opportunity for people to share their concerns and discuss them. This can be a self-healing mechanism for individuals.

Women had a greater and more diverse variety of social ties than men, as illustrated in Figure 5, when analyzing the effect of gender on modifying social relationships during a pandemic. Following the COVID-19 pandemic, both men and women have witnessed a drop in interpersonal connections, with women experiencing a higher decline. This disparity could indicate that women in this area are more vulnerable to the pandemic. A study published by the Lancet Commission on Women and Cardiovascular Diseases in 2021 also noted the importance of psychosocial factors such as depression and anxiety in the development of heart disease and the greater vulnerability of women in this area. The study found that women were more likely than men to experience social psychological damage such as chronic stress, grief, unemployment and lack of social support, which predisposed them to depression, anxiety and other mental health problems. Another cause of women’s vulnerability in mental health can be endogenous and hormonal issues (37).

On the other hand, it is possible that women were more active in caring for children and maintaining the family at home during the quarantine period than men, resulting in their isolation.

A 2020 study by Almeida et al. examined the effect of the Quaid pandemic on women’s mental health and found that post-partum pregnant women with abortions and women exposed to domestic violence were more likely to develop mental health problems in a pandemic. This indicates that this population needs special attention during a pandemic to reduce the burden of mental health problems (38).

Another study examining the management of women’s stress and lifestyle during the Quaid pandemic noted the effect of quarantine on reducing physical activity and increasing stress on increasing cardiovascular risk factors (39).

During the COVID-19 pandemic, the 18–24 age group was more constrained in interpersonal interactions than other age groups. This could be due to this age group’s enthusiasm and proficiency in using social media as a substitute for actual interactions during the pandemic. Because peer group communication is so vital in the formation of personality and social skills in this age group, it is critical to give extra attention to this group during pandemics. However, the pandemic has had less of an impact on the social relations of those aged 45 and up. This could be because this age group makes up the majority of the community’s employees, whose interpersonal ties are less harmed because of workplace communication.

The pandemic has resulted in a decline in interpersonal bonds among people of all socioeconomic levels. However, the findings demonstrate that this drop in interpersonal ties is not apparent in the middle class, as measured by income. One hypothesis is that, due to financial problems and fears of job instability, these people had more participation during the pandemic than other groups in society.

People with higher education (bachelor’s, master’s, and Ph.D.) had greater social contacts before and during the pandemic, but these interactions declined after the outbreak. In order to limit the danger of COVID-19, this might be done consciously in this subgroup. As a result, after the pandemic, those with lower levels of education in the community have not changed their interpersonal ties.

Individuals on both ends of the trust scale (total trust or absolutely without trust) have had no effect on their social connections as a result of the pandemic. This is likely due to distrustful people’s pessimism about news and prevention recommendations, and as a result, they do not follow these measures or do not believe in the risk of COVID-19 disease. They are likely to be reckless, misjudging the probability of the pandemic, and hence decided not to change their lifestyle.

According to Figure 5, the majority of people’s use of social media has remained unchanged in the aftermath of the pandemic. However, over time, during the pandemic, people’s participation in social media has increased, which has been the highest growth in the first survey. In other words, the average use of social media during a pandemic by individuals has increased on average. A study (40) looked into the psychological and social effects of the pandemic in the Najran City population, in Saudi Arabia. They discovered that during a pandemic, people’s use of social media increased and was linked to despair and anxiety. This result contradicted the findings of our study (in which the pattern of use of the majority of people has remained unchanged), which could be attributable to variations in the populations investigated, study tools, or analysis approach.

More women than men sought refuge on social media during the pandemic, which is to be expected given the further decline in their actual social interactions (mentioned above). The 18–24 age group has used social media more than others, and they are also the ones who have seen the largest drop in interpersonal interactions.

The number of persons who have declined to volunteer since the pandemic broke out has risen dramatically. However, there was no discernible difference in the amount of these activities before and after the pandemic. This finding can be interpreted as a result of people’s fear of developing or restrictions imposed during this time, such as quarantine. Individuals’ decreased empathy and compassion during the pandemic, on the other hand, could be considered as another interpretation. The rate of rejection has been higher in women, and this drop has been seen across practically all age groups, which is surprising given women’s perceived sensitivity. This difference is most likely owing to women’s vulnerability to mental health issues during a pandemic, which has hindered their willingness to volunteer. Prior to Pandemic, people with greater incomes and higher education had the most volunteer engagement, which decreased after the Pandemic. This discovery is significant because it can aid in identifying persons who are willing to take voluntary action. This shows that efforts encouraging people to participate more during the pandemic are needed. Figure 2D demonstrates that the majority of people (77.2%) did not experience employment insecurity during the pandemic compared to the previous, but they experienced increased job insecurity during the pandemic in the third survey. This can be read in light of changes in employment conditions and individual financial worries during the pandemic.

When reading the graph (Figure 7) used to model mental health prediction, it is important to note that some of the nodes and connections have had the largest impact on predicting mental health during a pandemic, as indicated in the image. People who have stayed at home during quarantine and used this method to prevent COVID-19, those who have not had any physical or psychiatric symptoms in the previous week, and those who are more comfortable with everyday contact are more confident that they will have fewer psychiatric problems during a pandemic, according to this model. This modeling aids in the identification of protective features that can be strengthened in individuals during future pandemics. On the other hand, it helps to identify vulnerable people with mental health problems. People having a history of medical issues and a history of past psychiatric problems, for example, appear to require more significant monitoring and preventive actions during a pandemic.

The utilization of machine learning techniques in data analysis in the realm of mental health issues is one of the study’s strengths. Also, analyzing the specifics of people’s social ties and how they changed over time (as influenced by the Pandemic), can lead to a deeper understanding of how people’s lifestyles changed during the pandemic. Given that more pandemics are almost likely to occur in the future, it is critical to use the information gathered to identify vulnerable people in each area and build effective preventative and treatment strategies. One of the study’s flaws is that changes in people’s social status can be impacted by a range of variables, some of which are ambiguous and have been neglected in this study. It is recommended that the findings of this study should be used in more specialized investigations in the field of mental health in vulnerable populations.

### Limitations and future research

This study only analyses the data for the duration of three months. Although this provides an indication of the impact of COVID-19 on mental health, it requires more comprehensive studies for a duration of 12 months to provide enhanced analysis of the impact. This study aimed to provide an indication of this impact on mental health.

This study provides the insight from the United States dataset. The model is representative of the majority of the ethnicities and influences in the United States due to the relatively high sample size and multi ethnic participation in the survey but would not be sufficiently representative of the other geographical areas, various cultures and social behavior.

In future studies, the work advances the use of explainable AI to forecast population-level mental health using survey data, making it generally applicable. The algorithms will be applied as a screening tool to identify people who require assistance, and subsequent research using more data and attributes may improve prediction accuracy. To effectively manage and avoid psychiatric comorbidities as populations continue to fight the pandemic, prediction models for screening and monitoring the effects of COVID-19 on mental health are essential.

On the other hand, this analysis would be expanded to other regions as well as other cultures to illustrate the relation between the COVID-19 impact on mental health and cultural behavior.

## Conclusion

This study analyzed the occurrence of mental illness among adults with and without a history of mental disease during the COVID-19 preventative limitations. Sample data was employed from the COVID-19 Impact Survey on April 20–26, 2020, May 4–10, 2020, and May 30–June 8, 2020 from United States Dataset. A total number of 8790, 8975, and 7506 adults participated in this study for April, May and June, respectively. Participants’ mental health evaluations were compared clinically by looking at the quantity and quality of their social ties before and during the pandemic using machine learning techniques. The result for each contributing feature has been analyzed separately in details. On the other hand, the influence of each feature was studied to evaluate the impact of COVID-19 on mental health. The overall result of our research indicates that people who had previously been diagnosed with any type of mental illness were most affected by the new constraints during the pandemic. These people were among the most vulnerable to mental illness due to the imposed changes in lifestyle. With the persistence of quarantine limitations, the prevalence of psychiatric issues grew. In the third survey, which was done under quarantine or house restrictions, mental health problems and acute stress reactions were substantially greater than in the prior two surveys. The findings of the study reveal that more focused messaging and support are needed for those with a history of mental illness throughout the implementation of restrictions.

## Data Availability

The original contributions presented in this study are included in the article, further inquiries can be directed to the corresponding author/s.
